# Parent-reported measure of repetitive behavior in Phelan-McDermid syndrome

**DOI:** 10.1186/s11689-021-09398-7

**Published:** 2021-11-05

**Authors:** Siddharth Srivastava, Emma Condy, Erin Carmody, Rajna Filip-Dhima, Kush Kapur, Jonathan A. Bernstein, Elizabeth Berry-Kravis, Craig M. Powell, Latha Soorya, Audrey Thurm, Joseph D. Buxbaum, Mustafa Sahin, A﻿lexander Kolevzon, on behalf of Developmental Synaptopathies Consortium, Mustafa Sahin, Alexander Kolevzon, Joseph D. Buxbaum, Elizabeth Berry Kravis, Latha Soorya, Audrey Thurm, Craig Powell, Jonathan A. Bernstein, Simon Warfield, Kira Dies, Paige Siper, Ellen Hanson, Jennifer M. Phillips

**Affiliations:** 1 Department of Neurology, Rosamund Stone Zander Translational Neuroscience Center, Boston Children’s Hospital, Harvard Medical School, Boston, MA USA; 2 Neurodevelopmental and Behavioral Phenotyping Service, National Institute of Mental Health, National Institutes of Health, Bethesda, MD USA; 3 Department of Pediatrics, Stanford University School of Medicine, Stanford, CA USA; 4 Department of Pediatrics, Rush University Medical Center, Chicago, IL USA; 5 Department of Neurological Sciences, Rush University Medical Center, Chicago, IL USA; 6 Department of Biochemistry, Rush University Medical Center, Chicago, IL USA; 7 Department of Neurobiology, University of Alabama at Birmingham School of Medicine, Birmingham, AL USA; 8 Civitan International Research Center, University of Alabama at Birmingham, Birmingham, AL USA; 9 Department of Psychiatry, Rush University Medical Center, Chicago, IL USA; 10 Seaver Autism Center for Research and Treatment, Mount Sinai School of Medicine, New York, NY USA; 11 Department of Psychiatry, Icahn School of Medicine at Mount Sinai, New York, NY USA; 12 Department of Genetics and Genomic Sciences, Mount Sinai School of Medicine, New York, NY USA; 13 Department of Neuroscience, Mount Sinai School of Medicine, New York, NY USA; 14 F.M. Kirby Neurobiology Center, Boston Children’s Hospital, Harvard Medical School, Boston, MA USA

**Keywords:** SHANK3, Intellectual disability, 22q13 deletion, Repetitive behavior, Stereotypy, Autism

## Abstract

**Background:**

Phelan McDermid syndrome (PMS) is a neurogenetic condition associated with a high prevalence of intellectual disability (ID) and autism spectrum disorder (ASD). This study provides a more comprehensive and quantitative profile of repetitive behaviors within the context of ID seen with the condition.

**Methods:**

Individuals age 3–21 years with a confirmed PMS diagnosis participated in a multicenter observational study evaluating the phenotype and natural history of the disorder. We evaluated data collected from this study pertaining to repetitive behaviors from the Repetitive Behavior Scales-Revised (RBS-R).

**Results:**

There were *n* = 90 participants who were part of this analysis. Forty-seven percent (*n* = 42/90) were female, and the average age at baseline evaluation was 8.88 ± 4.72 years. The mean best estimate IQ of the cohort was 26.08 ± 17.67 (range = 3.4–88), with *n* = 8 with mild ID (or no ID), *n* = 20 with moderate ID, and *n* = 62 with severe-profound ID. The RBS-R total overall score was 16.46 ± 13.9 (compared to 33.14 ± 20.60 reported in previous studies of ASD) (Lam and Aman, 2007), and the total number of items endorsed was 10.40 ± 6.81 (range = 0–29). After statistical correction for multiple comparisons, IQ correlated with the RBS-R stereotypic behavior subscale score (*r*_*s*_ = − 0.33, unadjusted *p* = 0.0014, adjusted *p* = 0.01) and RBS-R stereotypic behavior total number of endorsed items (*r*_*s*_ = − 0.32, unadjusted *p* = 0.0019, adjusted *p* = 0.01). IQ did not correlate with any other RBS-R subscale scores.

**Conclusions:**

The RBS-R total overall score in a PMS cohort appears milder compared to individuals with ASD characterized in previous studies. Stereotypic behavior in PMS may reflect cognitive functioning.

## Background

Phelan-McDermid syndrome (PMS), a genetic cause of intellectual disability (ID) and autism spectrum disorder (ASD), is a condition of abnormal synaptic transmission caused by a pathogenic variant affecting *SHANK3*, either through an intragenic variant or a 22q13 deletion. Affected individuals present with a broad spectrum of somatic and neurobehavioral features, including facial and systemic anomalies, global developmental delay leading to ID often in the severe or profound range, absent or delayed speech, and generalized hypotonia [1]. Reports thus far indicate that almost all individuals with PMS meet diagnostic criteria for ID. More than 50% of individuals with PMS meet diagnostic criteria for ASD [2, 3] and up to 2% of individuals with ASD have *SHANK3* haploinsufficiency [4, 5]. Given the marked presence of severe to profound ID in PMS and the difficulties of diagnosing ASD in this context [6], further investigation of how ASD symptoms manifest and relate to cognitive ability in PMS is warranted.

Prior studies of the behavioral phenotype of PMS have focused broadly on the behavioral profile, including ASD symptomatology. Despite the caveat that many individuals with PMS have mental ages below the minimum required for ASD measures to be considered valid [7], several studies have implemented such measures in this population. In one study that administered the Autism Diagnostic Interview-Revised (ADI-R) to a group that did not have mental ages established to determine validity of the measure, 90% of individuals with PMS had deficits in social-communication above the diagnostic cutoff established for this measure, while 55% were above the cutoff for restricted, repetitive patterns of behavior, interests, or activities [2]. However, scores on the ADI-R did not predict a clinical diagnosis of ASD in a regression model [2]; instead, ASD diagnosis was predicted by impaired socialization scores on the Vineland Adaptive Behavior Scales, Second Edition (Vineland-II). There is a great need to better characterize behaviors that are characteristic of ASD and ID to a lesser extent [8, 9] in PMS, especially due to the severity of ID in this population. While the need to appropriately measure ASD symptoms in genetic conditions characterized by ID has been emphasized, much of this critique has focused on the social-communication deficits in these conditions and not repetitive behaviors. Although previous studies suggest a relationship between repetitive behaviors and IQ in ASD [10–13] and other conditions associated with ASD and ID, such as fragile X syndrome (FXS) [14], it will be valuable to determine if this relationship is maintained in individuals with severe to profound ID or if there is a unique profile of repetitive behaviors in PMS.

Repetitive behaviors may impact learning and social development [15, 16] and, in the case of self-injury, threaten the health and safety of affected children through risk of physical harm and infection [17]. Thus, repetitive behaviors are a target for treatment not only for ASD [18] but also for conditions associated with ID [19–21] more generally. Better characterization of the restricted and repetitive behavior (RRB) profile of PMS is needed in order to develop hypotheses regarding which interventions may be most effective in this population.

Using ASD assessment tools, some studies have begun to highlight important differences between the repetitive behavior profile in idiopathic ASD and specific genetic conditions such as PMS. Certain repetitive behaviors, namely stereotyped behaviors, are commonplace in both idiopathic ASD and in conditions characterized by varying degrees of ID [8, 22]. In PMS, other repetitive behaviors occur less frequently, if at all [3]. For example, unusual preoccupations, resistance to changes in the environment, and unusual attachments to objects are less commonly reported in PMS based on the ADI-R [3]. However, ASD assessment tools such as the ADI-R and Autism Diagnostic Observation Schedule (ADOS) do not capture the full profile of repetitive behaviors that present in these populations. Instead, assessment tools such as the Repetitive Behavior Scale-Revised (RBS-R) [23] or the Repetitive Behavior Questionnaire [24] allow for more thorough characterization of these heterogenous behaviors, which vary in prevalence and profile across other genetic syndromes [22]. Additionally, through measures such as the RBS-R, RRBs have been shown to psychometrically fall into subtypes, which are thought to comprise separate constructs. Often these are divided into higher-order and lower-order RRBs, which are proposed to have different underlying etiologies, but can be further split into more specific categories of behavior (see [25, 26] for a summary of these various factor structures). With this in mind, profiles of RRBs in ASD and ID have been described through these different RRB subtypes to better elucidate the variable profile of RRBs presented. One such study recently utilized the Repetitive Behavior Questionnaire as part of survey data collection comparing a sample of PMS to fragile X syndrome, Down syndrome, and idiopathic ASD, finding lower levels of total repetitive behavior in PMS compared to idiopathic ASD and fragile X. This difference was largely attributable to relatively lower compulsive behavior and insistence on sameness in PMS compared to these disorders, while individuals with PMS exhibited comparable levels of repetitive motor movement [27]. However, as a survey, this study was limited in its ability to factor in level of ID in its analysis, which could account for the consistency in stereotyped behaviors seen across diagnostic groups. Indeed, previous studies indicate that IQ is specifically associated with stereotypic behaviors [10–13]. For this reason, further exploration of the profile of repetitive behavior in PMS using a measure of RRB as part of a comprehensive behavioral phenotyping protocol is warranted.

The current study aimed to characterize repetitive behaviors in PMS, as well as the cognitive ability that contextualizes them, using parent-reported measures and developmental testing. In 2015, the Developmental Synaptopathies Consortium was established to initiate a large multicenter study with a central goal of tracking the natural history of PMS and discovering potential phenotypic and genotypic factors that contribute to diverse patient outcomes. In this analysis, we focused our efforts on analyzing data from the RBS-R, a validated parent-reported instrument of repetitive behavior, to characterize repetitive behaviors in this population. Given that stereotyped behaviors are related to ID, and PMS is characterized by severe ID, we expect that stereotyped behaviors will be elevated in PMS, but other types of repetitive behavior which have not been related to level of ID will be less severe (e.g., compulsive behavior, ritualistic and sameness behaviors). Additionally, based on prior data linking stereotyped behaviors to IQ [10–13], we hypothesize that in PMS, the degree of repetitive behaviors, and particularly stereotyped behaviors, is directly related to ID severity.

## Methods

### Study participants

We performed a cross-sectional analysis of baseline data from 90 individuals with PMS enrolled in a prospective, multi-site, observational, cohort study evaluating the phenotype and natural history of PMS (ClinicalTrials.gov NCT02461420). English speaking males or females, ages 3–21 years, with pathogenic chromosomal deletions or pathogenic variants causing happloinsufficiency of the *SHANK3* gene, were eligible for the study. Participant recruitment was from PMS clinics across the USA, in coordination with the PMS Foundation. Participants underwent in person assessments by the study team at the various sites of this multisite study, after appropriate consent took place on this study approved by a centralized IRB at Boston Children’s Hospital. Assessments were conducted by neuropsychologists and other members qualified to administer assessments as designated below.

### Behavioral assessments

#### Repetitive Behavior Scale–Revised (RBS-R)

Caregivers completed the RBS-R [23], a caregiver-reported questionnaire with 43 items focusing on restricted and repetitive behaviors. Each item has a possible integer score from 0 to 3: 0 = the behavior does not occur; 1 = the behavior occurs and is a mild problem; 2 = the behavior occurs and is a moderate problem; 3 = the behavior occurs and is a severe problem. The 43 items in the instrument span six subscales: stereotyped behavior subscale, self-injurious behavior subscale, compulsive behavior subscale, ritualistic behavior subscale, sameness behavior subscale, and restricted behavior subscale. Each of the six subscales has two corresponding values: one for the total number of subscale items endorsed, and one for the sum of all the scores within the subscale. Importantly, the original version of this measure, the Repetitive Behavior Scale, included a validation sample of individuals with ID. Specifically, the sample included an ASD + ID and ID-alone comparison group, each noted to consist of a majority of individuals with severe or profound ID, with only two participants per group with IQ outside of the severe to profound ID range [8].

Lam and Aman (2007) validated the RBS-R in individuals with ASD ages 3–48 years (whose parents/caretakers were members of the South Carolina Autism Society) and refactored the original six subscale instrument into five subscales: stereotypic behavior subscale, self-injurious subscale, compulsive subscale, ritualistic/sameness subscale, restricted interests subscale [28]. This study did not indicate the level of ID in their sample, but did note the education placement for their sample (57.7% “special class in a regular school;” 16.6% “regular class in a regular school;” 14.7% “special school;” 9.4% “other”). In this refactoring, five single items did not load into one of these five subscales, resulting in a total of 38 single items out of the 43 in the original RBS-R. We use this refactored formulation of the RBS-R in our analysis. The range of possible values for each subscale score is 0 to 3 multiplied by number of items within that subscale. There are overall scores for the total number of items endorsed (possible range = 0–38 in the refactored version) and the sum of all the item scores (overall total score; possible range = 0–114 [38 items × max score of 3] in the refactored version).

#### Vineland Adaptive Behavior Scales–Second Edition (Vineland-II)

Caregivers were interviewed using the Vineland Adaptive Behavior Scales, Second Edition (Vineland-II), which is a standardized tool to evaluate adaptive behavior pertaining to the domains of communication, socialization, daily living skills, and motor skills [29]. Domain scores generate an overall adaptive behavior composite standard score. The study used the interview form of the assessment.

#### Autism spectrum disorder consensus diagnosis

Individuals received a diagnosis of ASD based on Diagnostic and Statistical Manual for Mental Disorders, Fifth Edition criteria [30], informed by the ADI-R [31], Autism Diagnostic Observation Schedule, Second Edition (ADOS-2) [32], and clinical judgment, which included ratings of clinical certainty.

#### IQ

For each participant, we generated a best estimate IQ based on standard scores on IQ tests or ratio IQ estimates in those whose scores on cognitive tests were out-of-range. We used a hierarchy of tests, including the Mullen Scales of Early Learning [33] and the Stanford Binet [34]. This framework has been previously established for use in individuals with severe-profound ID [7]. IQ scores from the Stanford Binet were used for participants who could achieve a basal score on this measure; developmental quotients (mental age divided by chronological age) were used for participants who were administered the Mullen Scales of Early Learning due to the high rate of this measure being administered out-of-age-range (above chronological age of 5 years 8 months) or, if in age range, receiving a standard score at the basal for the measure. For the purpose of this study, ID groupings were categorized as (1) mild ID (best estimate IQ ≥ 50), (2) moderate ID (best estimate IQ of ≥ 35 and < 50), (3) severe-profound ID (best estimate IQ < 35).

### Statistical analysis

For the analysis, we excluded individuals with incomplete baseline behavioral assessments (specifically missing best estimate IQ, RBS-R, and Vineland-II [apart from the motor domain]). We used descriptive analyses to present means and frequencies (standard deviations following means are denoted with plus-minus sign).

First, to provide a description of the repetitive behavior profile in PMS, descriptive statistics on the number of RBS-R items endorsed (score of > 0), RBS-R subscale scores, and RBS-R total score in the PMS cohort are provided. Next, to address the hypothesis that RRB severity in PMS is reflective of level of cognitive functioning, we conducted a series of Spearman rank-order correlations between RBS-R subscale scores and IQ. We used Wilcoxon rank-sum test to examine IQ across sex; logistic regression model to compare ASD diagnosis with IQ; and Spearman rank correlation to correlate IQ with age and VABS-II scores. To account for multiple comparisons, we used Benjamini-Hochberg (BH) false discovery rate procedure separately for each instrument where the total number of statistical comparisons was ≥ 10. We chose *q* = 0.05. The ± sign indicates standard deviation.

## Results

### Demographic and clinical characteristics

There were 90 participants with complete behavioral assessment data who were part of this analysis. Forty-seven percent (*n* = 42/90) were female, and the average age at baseline evaluation was 8.88 ± 4.72 years. In terms of race, 83% of participants were white, 9% were Asian, 3% were African American, 1% were American Indian or Alaskan native, and 3% had unknown/unreported race. The mean best estimate IQ of the cohort was 26.08 ± 17.67 (range = 3.4–88), with *n* = 8 with mild ID (or higher), *n* = 20 with moderate ID, and *n* = 62 with severe-profound ID. Fifty-nine percent of the cohort (*n* = 51/86) had a diagnosis of ASD; the highest rate of ASD occurred in the severe-profound ID group (73%; *n* = 43/59), and the lowest rate (14%; *n* = 1/7) occurred in the mild ID or higher group. See Table 1.

**Table 1:** Demographic and developmental characteristics of the cohort. Statistic pertains to comparison of variable versus quantitative IQ (not ID group). We used Wilcoxon rank-sum test to examine IQ across sex; Spearman rank correlation to correlate IQ with age and VABS-II scores; and logistic regression model to compare ASD diagnosis with IQ.

	Mild ID			Moderate ID			Severe profound ID			Entire cohort			Statistic	***p***-value
	Mean	SD	N	Mean	SD	N	Mean	SD	N	Mean	SD	N		
Gender (% Female)	0.62		8	0.55		20	0.42		62	0.47		90	W = 1194	0.13
Age at evaluation (years)	7.48	1.32	8	11.69	5.03	20	8.15	4.58	62	8.88	4.72	90	r_s_ = -0.08	0.45
VABS-II Adaptive Behavior Composite Standard Score	74.12	7.94	8	60.5	10.06	20	44.56	9.43	62	50.73	13.59	90	r_s_ = 0.84	<0.0001
VABS-II Communication Standard Score	78	8.12	8	61.15	11.65	20	42.37	9	62	49.71	15.12	90	r_s_ = 0.84	<0.0001
VABS-II Socialization Standard Score	82.38	12.59	8	64.6	12.43	20	50.47	10.71	62	56.44	14.98	90	r_s_ = 0.76	<0.0001
VABS-II Daily Living Skills Standard Score	65.88	16.62	8	61.05	12.22	20	45.32	10.88	62	50.64	14.13	90	r_s_ = 0.75	<0.0001
VABS-II Motor Standard Score	71.29	10.03	7	69.15	10.54	13	53.1	7.9	50	57.9	11.45	70	r_s_ = 0.75	<0.0001
ASD (%)	0.14		7	0.35		20	0.73		59	0.59		86	IQ coefficient: estimate = -0.07, std error = 0.02	<0.0001

### RBS-R item-level prevalence and severity

The mean total number of items endorsed was 10.4 ± 6.81 (range = 0–29), approximately 26% of the items using the 38-item Lam and Aman (2007) scoring [28]. For each participant, the average number of RBS-R subscales containing at least one endorsed item was 3.4 ± 1.5 (range = 0–5). Among the items in the refactored RBS-R, there were four items associated with a prevalence (as determined by whether the item score was either 0 or at least 1) above 50%: item 3 [68%; “hand/finger (flaps hands, wiggles or flicks fingers, claps hands, waves or shakes hand or arm)”]; item 36 [64%; “likes the same CD, tape, record, or piece of music played continually; likes same movie/video or part of movie/video”]; item 37 [56%; “resists changing activities; difficulty with transitions”]; and item 5 [52%; “object usage (spins or twirls objects, twiddles or slaps or throws objects, lets objects fall out of hands)”]. RBS-R item 3 and item 5 are within the stereotyped behavior subscale; item 36 is within the restricted interests subscale; and item 37 is within the ritualistic/sameness subscale.

### RBS-R total and subscale scores

**Fig. 1:**
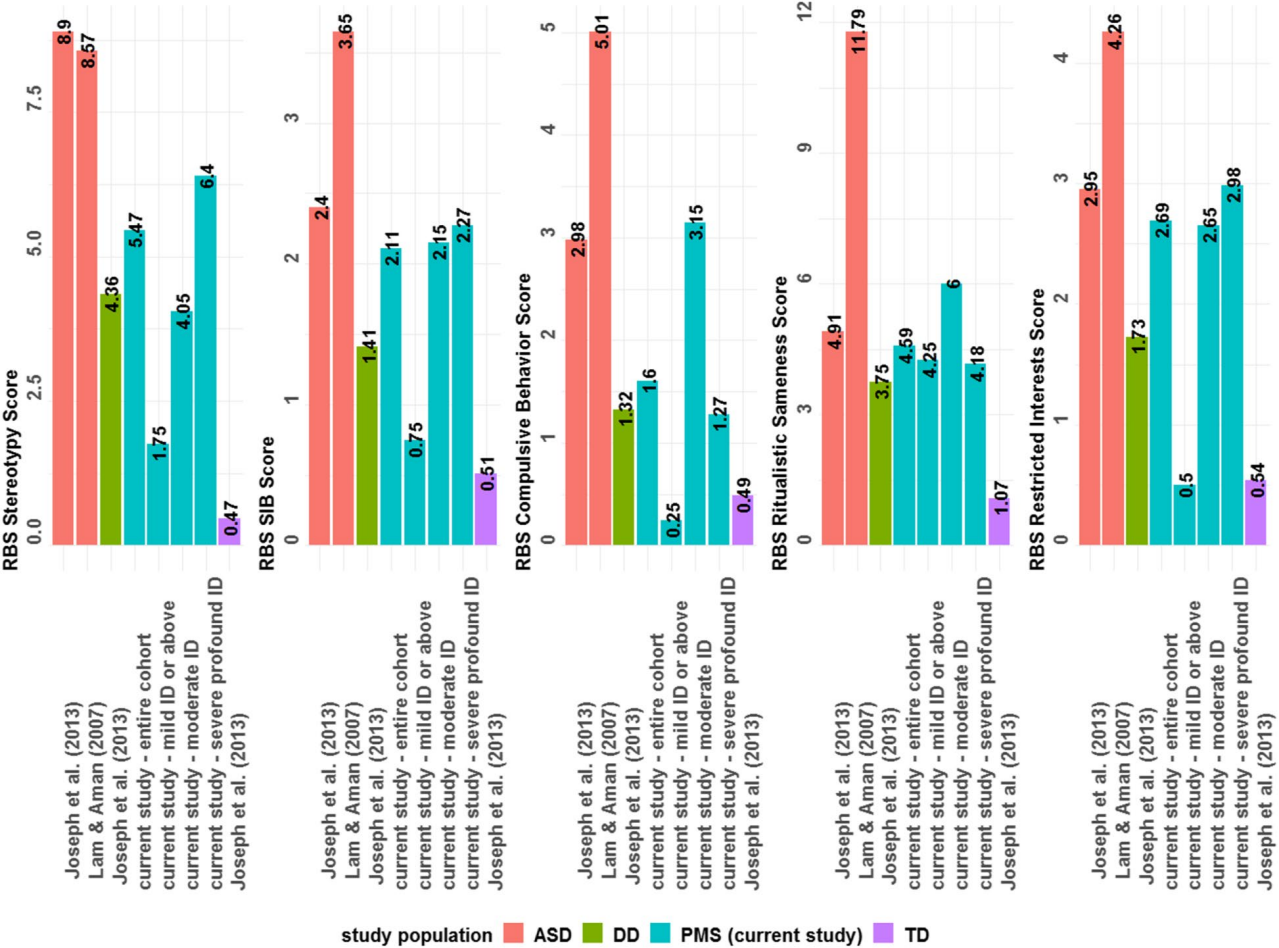
Mean RBS-R subscales scores in this cohort compared to data from prior published cohorts. ASD = autism spectrum disorder; DD = developmental delay; TD = typically developing

On the RBS-R, the mean total overall score was 16.46 ± 13.9. Additionally, the RBS-R subscale scores from this PMS sample are summarized alongside scores previous studies of individuals with and without ID and/or ASD to provide context for how RRBs in PMS compare to other conditions (Fig. 1). Two prior cohorts included in the figure have focused on children with ASD [*n* = 267–307, mean IQ not reported [28]; *n* = 128, mean IQ = 59 [25]]; developmental delay [*n* = 44, mean IQ = 61 [25]]; and typical development [*n* = 59, mean IQ = 109 [25]]. Generally, scores in the PMS cohort appear elevated across all subscales compared the typical development cohort from Joseph et al. (2013) [25] but lower than the ASD groups from Joseph et al. (2013) [25] and Lam and Aman (2007) [28].

### Relationship between RBS-R scores and ID

We conducted Spearman’s rank order correlations comparing IQ versus RBS-R subscale scores, subscale number of endorsed items, overall total score, and overall total number of endorsed items. After statistical correction for multiple comparisons, IQ correlated with the RBS-R stereotypic behavior subscale score (*r*_*s*_ = − 0.33, unadjusted *p* = 0.0014, adjusted *p* = 0.01) and RBS-R stereotypic behavior total number of endorsed items (*r*_*s*_ = − 0.32, unadjusted *p* = 0.0019, adjusted *p* = 0.01), but not with any other RBS-R subscale scores. See Table 2. In a binomial generalized linear model of ASD diagnosis versus RBS-R stereotypic behavior subscale score, with IQ as a co-variate, coefficient of the RBS-R term was not statistically significant (estimate = 0.11, *p* = 0.061).

**Table 2:** RBS-R subscale scores, subscale number of endorsed items, overall total score, and overall total number of endorsed items, stratified by ID severity. Statistic pertains to comparison of variable versus quantitative IQ (not ID group). We used Spearman rank correlation to correlate IQ with variable.

	Mild ID			Moderate ID			Severe-profound ID			Entire cohort			Statistic	***p***-value	Adjusted ***p***-value
	Mean	SD	N	Mean	SD	N	Mean	SD	N	Mean	SD	N			
Stereotyped behavior subscale score	1.75	2.19	8	4.05	5.1	20	6.4	4.76	62	5.47	4.88	90	r_s_ = -0.33	0.0014	0.011
Stereotyped behavior subscale number of endorsed items	1.62	2	8	2.6	2.33	20	4.02	2.36	62	3.49	2.45	90	r_s_ = -0.32	0.0019	0.011
Self-injurious behavior subscale score	0.75	1.16	8	2.15	2.64	20	2.27	2.63	62	2.11	2.55	90	r_s_ = -0.18	0.10	0.23
Self-injurious behavior subscale number of endorsed items	0.75	1.16	8	1.45	1.57	20	1.48	1.54	62	1.41	1.52	90	r_s_ = -0.15	0.17	0.25
Compulsive subscale score	0.25	0.46	8	3.15	3.51	20	1.27	1.99	62	1.6	2.48	90	r_s_ = 0.16	0.14	0.24
Compulsive subscale number of endorsed items	0.25	0.46	8	2.05	1.9	20	0.73	1.09	62	0.98	1.4	90	r_s_ = 0.19	0.07	0.20
Ritualistic/sameness subscale score	4.25	6.14	8	6	4.83	20	4.18	5.86	62	4.59	5.66	90	r_s_ = 0.07	0.50	0.50
Ritualistic/sameness subscale number of endorsed items	2.88	3.23	8	4.3	3.1	20	2.65	2.79	62	3.03	2.94	90	r_s_ = 0.09	0.39	0.43
Restricted subscale score	0.5	1.07	8	2.65	2.39	20	2.98	2.63	62	2.69	2.56	90	r_s_ = -0.21	0.04	0.17
Restricted subscale number of endorsed items	0.38	0.74	8	1.65	1.14	20	1.58	1.08	62	1.49	1.11	90	r_s_ = -0.13	0.24	0.31
Total score	7.5	9.18	8	18	14.92	20	17.11	13.74	62	16.46	13.85	90	r_s_ = -0.16	0.13	0.24
Total number of endorsed items	5.88	5.74	8	12.05	7.94	20	10.45	6.38	62	10.4	6.81	90	r_s_ = -0.10	0.37	0.43

## Discussion

On average, there is a full spectrum of repetitive behaviors in PMS relative to previous reports of typical development [25], but questions remain about whether the repetitive behavior profile in PMS is different from that of other neurodevelopmental disorders, and whether the severity of RRBs seen in PMS is attributable to the profound ID seen in this population. The RBS-R total overall score was 16.46 ± 13.85, which appears milder compared to individuals with ASD that have been characterized in previous studies. In participants with ASD (whose data served as the basis for the RBS-R refactoring used in this analysis), the mean (refactored) RBS-R total score was 33.14 ± 20.6 [28], nearly double the total RBS-R score in our cohort. However, when subscale scores differences between the Lam and Aman (2007) ASD group [28] and the PMS sample are compared (where *%* change = (PMS – ASD)/ASD), it is evident that the difference in total score could be driven largely by a relative absence of ritualistic/sameness behaviors (∆ = − 7.2, % change = − 61%) and compulsive behavior (∆ = − 3.4, *%* change = − 68%) in our PMS sample (Fig. 1). The other subscales scores were also lower in PMS, but not to the same extent in stereotyped behavior (∆ = − 3.1, % change = −36%), self-injurious behavior (∆ = − 1.5, % change = − 42%), and restricted interests (∆ = − 1.6, % change = − 37%).

Studies of other genetic disorders with a high prevalence of ASD and ID, such as fragile X syndrome (FXS), have shown elevated RBS-R total scores relative to the Lam and Aman (2007) ASD group (though it is important to note that these studies used the original, non-refactored RBS-R). These studies show higher total scores on the RBS-R compared to our PMS cohort (FXS alone = 20.5 ± 14.5, FXS + ASD = 27.1 ± 17.0 [35]; FXS adolescents = 27.7 ± 20.35, FXS adults = 25.2 ± 19.10 [36]). Questions remain as to why the scores in the present PMS cohort appear much lower than another genetic disorder characterized by ID and ASD.

One possibility is that PMS simply has a different RRB profile than ASD and other genetic conditions characterized by ID; however, it is difficult to draw this conclusion using the presented data without RBS-R profiles from other conditions to compare to our PMS group. Prior data may provide some insight. For example, a study comparing the phenotype of PMS to FXS, Down syndrome, and idiopathic ASD showed lower overall rates of repetitive behavior in PMS compared to FXS and idiopathic ASD on the Repetitive Behavior Questionnaire (RBQ) [27]. Item-level data were not examined, but RBQ subscales indicated that individuals with PMS had lower insistence on sameness and compulsive behavior subscale scores than idiopathic ASD and, in the case of insistence on sameness, FXS. Another study examined seven different genetic conditions (Angelman, Cornelia de Lange, Cri-du-Chat, Fragile X, Prader-Willi, Lowe, and Smith-Magenis syndromes), three of which (Cri-du-chat, Smith-Magenis, and Prader-Willi syndromes) were noted to display a relatively specific RRB profile at the item-level using the RBQ [22]. Those findings are not directly comparable to the data in the present paper because a different RRB measure was used, and PMS was not part of this analysis. However, it is notable that the Angelman syndrome profile was described as having “a lower level of specificity on most forms of repetitive behavior” across all domains, with a lower total score on the RBQ than the other genetic conditions. Angelman syndrome was the only syndrome in the study that was characterized by severe-profound ID, as is PMS (the other syndromes included wider IQ ranges encompassing mild and moderate ID). When item-level data within our PMS sample were examined for rates of endorsement, only 4 of the items were endorsed at a rate >= 50%, perhaps indicating that a “lower level of specificity” is also being displayed in PMS.

Though these neurogenetic developmental disorders are often characterized by ID, the present PMS sample is characterized by severe to profound ID evidenced by an average IQ of 26.08, whereas the FXS groups in the aforementioned studies had average IQs of 41 and 47, respectively. The differences in RBS-R score could be due to this difference in cognitive ability. Indeed, the relative absence of ritualistic/sameness behaviors noted in the PMS sample would be consistent with this theory, as “higher-order” behaviors are shown to be more prevalent in those with higher IQ [13] and are endorsed in populations with less severe ID, such as FXS [14]. In severe to profound ID, certain RBS-R items may have little variability or poor validity, as the scale has not been thoroughly validated in this population. Indeed, a recent FXS study indicated that specific items on the RBS-R produced the differences in subscale scores between diagnostic groups (i.e., FXS-alone versus FXS + ASD), indicating that these items may not maintain measurement invariance between diagnostic groups (e.g., ID-alone and ID+ASD groups) [14]. This area warrants further investigation. Another possible explanation for this discrepancy is that families/caretakers completing the RBS-R instrument may be answering with respect to what they believe are the norms for *other children/adults with PMS,* while in at least the aforementioned studies in FXS, families were instructed to rate relative to a typical child/adult.

Among repetitive behaviors, however, stereotypies are relatively more severe compared to other types of repetitive behaviors in PMS. Specifically, on the RBS-R, the prevalence of hand/finger stereotypies (item 3) was the highest. The severity of stereotyped behaviors, represented by the RBS-R stereotyped behavior, correlated significantly with IQ, but not ASD diagnosis. In support of this notion, prior studies have established a relationship between lower-order repetitive behaviors, such as stereotypic behavior, and lower IQ [10–13]. With respect to the refactored RBS-R used for this analysis, lower-order repetitive behaviors comprise the self-injurious behavior, stereotypic behavior, and restricted behavior subscales, while higher-order repetitive behaviors apply to the compulsive behavior and ritualistic/sameness behavior subscales. These groupings align with the marked difference in change scores noted above, because although PMS showed lower scores across all RBS-R subscales, a greater difference is apparent between the PMS cohort and Lam and Aman’s (2007) ASD sample [28] in higher-order RRB subscales than in the lower-order RRB. However, only the RBS-R stereotypic behavior subscale score correlated with IQ in PMS. Again, this discrepancy may be due in part to the fact our cohort was heavily imbalanced toward severe to profound ID with a significantly reduced range of IQ scores overall, potentially limiting the types of behaviors reflected in the other RRB subscales, even those that are considered lower-order behaviors. For example, on the Lam and Aman (2007) [28] restricted behavior subscale, item 40 reads: “fascination, preoccupation with one subject or activity (e.g., trains, computers, weather, dinosaurs)”, with the definition further specifying a “limited range of focus, interest or activity.” This item may require a certain level of cognitive ability (e.g., attention) for a parent to endorse a score above 0 and could account for the lower scores on this subscale in a population with severe to profound ID, such as PMS. It is possible that the relative decrease in RRB severity in the present study is attributable to the level of severe to profound ID seen in PMS; however, a sample of individuals with a wider range of ID would be better suited to answer this question and should be considered in future research.

One other way to investigate the discrepancy of RBS-R scores in PMS versus other neurodevelopmental disorders is through the investigation of neurobiological pathways underlying types of repetitive behavior using neuroimaging. Most of this neuroimaging work has only been conducted at the level of RRB total severity, but there is evidence that the same systems may be involved across these disorders. In young children with ASD, neuroanatomical changes in the basal ganglia and thalamus may play a role in the underlying pathophysiology of repetitive behaviors [37]. Additionally, a review of imaging studies in neurodevelopmental disorders indicates that RRBs relate not only to basal ganglia alterations, but also frontotemporal areas and the cerebellum [38]. The basal ganglia (caudate, putamen, and pallidum) and cerebellum have been explored in a small cohort of individuals with PMS, with the basal ganglia regions shown to be smaller in patients relative to controls [39]. Furthermore, a negative relationship between cerebellar volume and RBS-R total score was found in the PMS group, extending the relationship between the cerebellum and RRBs from the ASD and ID literature to PMS. While the frontotemporal areas found in previous ASD studies were not implicated in the PMS group [39], this may be a result of the differences in the types of RRBs displayed in each group as described in the present study. If PMS shows relatively less severe higher-order RRB than ASD as evidenced in our study, then brain regions involved in the etiology of higher-order RRB may be implicated in an ASD cohort but not a PMS cohort. This may account for the relation between RRB and frontotemporal areas in ASD [38] which was not reported in PMS. However, brain regions related to RRB scores in *both* the PMS and ASD samples, namely the cerebellum and basal ganglia, may be associated with the presence of the behaviors that are seen across both disorders, such as stereotypic behaviors. Not only is this consistent with the findings presented in our study, but also maintains face validity because of the cerebellum’s role in sensorimotor processes and established relationship with the basal ganglia [40]. Additionally, *Shank3* deficient mice exhibit repetitive grooming (considered a behavioral assay of RRB), deficits in striatal and cortico-striatal synapses [41–44], and altered excitatory/inhibitory signaling [42–47]. The precise role of these neural factors in the formation of repetitive behaviors in humans with PMS remains largely untested and an important area for future research.

The results of this study provide a comprehensive assessment of the repetitive behavior profile in PMS; however, there are a number of limitations to consider. While this study follows participants for biannual assessments over three years, the present study focuses on a cross sectional analysis of baseline data only. To maximize the potential understanding of the repetitive behavior profile in PMS, future analyses will examine longitudinal data not presented herein. Additionally, the PMS sample in the present study is notably different not only in the level of ID, but also in its sex composition compared to ASD samples from previous studies. Recent studies have shown that there may be differences in endorsement of RRB items related to sex in ASD populations [48], and presumably this may carry over to other conditions. With this in mind, future studies of RRB should potentially control for sex differences when comparing genetic conditions to idiopathic ASD samples. In addition, the repetitive behavior assessment was limited to the RBS-R. Caregiver-report instruments in particular may not be ideal as parents may under-report symptoms by comparing their child to what is expected in PMS rather than to the normative population. In the future, standardized training for families on completing rating forms and the use of objective measurements of repetitive behaviors may be helpful to improve the validity of results. For example, recent advances in wearable biosensors and machine learning have allowed for automatic detection of RRB occurrences [49] so that objective measurement of stereotypic behaviors might be feasible in future studies. Finally, in our neurodevelopmental phenotyping, we did not analyze data from ADOS-2 or ADI-R, given that a majority of our cohort had severe-profound ID and a mental age < 18 months, below the threshold for which these measures are valid. In fact, a recent analysis of data collected from this cohort on the Social Responsiveness Scale confirmed the lack of validity for at least this measure of ASD in this population [50].

## Conclusions

In sum, the RRB profile seen in PMS, a condition characterized by severe to profound ID and co-occurring ASD, appears milder than RRB severity as previously reported in ASD samples. As was hypothesized, the most severe RRB subtype in this sample appears to be stereotypic movements, which was related to level of cognitive functioning. In addition to providing insight about the behavioral profile of PMS, these findings indicate that perhaps certain types of RRB should be preferentially targeted when investigating future etiologies and therapeutics for behaviors associated with PMS.

## Data Availability

We entered data in a web-based system created and maintained by the Data Management and Coordinating Center at the University of South Florida meeting Health Insurance Portability and Accountability Act privacy regulations. Clinical data presented here are deposited in the National Database for Autism Research, an NIH-funded data repository which stores and shares data pertaining to autism spectrum disorder with qualified researchers.
